# Maternal PM_10_ Exposure Increases Risk for Spina Bifida: A Population-Based Case-Control Study

**DOI:** 10.3389/fpubh.2021.695192

**Published:** 2021-07-21

**Authors:** Huan Li, Yan-Hong Huang, Jing Li, Shu Liu, Yan-Ling Chen, Li-Li Li, Cheng-Zhi Jiang, Zong-Jiao Chen, Na Li

**Affiliations:** ^1^Department of Obstetrics and Gynecology, Shengjing Hospital of China Medical University, Shenyang, China; ^2^Department of Ophthalmology, Shenyang Women's and Children's Hospital, Shenyang, China; ^3^Department of Science and Education, Shenyang Maternity and Child Health Hospital, Shenyang, China; ^4^Department of Atmospheric Environment Monitoring, Liaoning Eco-Environmental Monitoring Center, Shenyang, China; ^5^Liaoning Women and Children's Health Hospital, Shenyang, China; ^6^Department of Children's Health Prevention, Shenyang Maternity and Child Health Hospital, Shenyang, China; ^7^School of Environmental and Chemical Engineering, Shenyang Ligong University, Shenyang, China

**Keywords:** PM_10_, spina bifida, birth defects, air pollution, particulate matter, case-control study

## Abstract

Limited studies have focused on the impact of ambient air pollution on spina bifida. A population-based case-control study was conducted in Liaoning Province, China to assess the associations between maternal PM_10_ exposures in various exposure windows and spina bifida risk. Data on spina bifida cases born between 2010 and 2015 were available from the Maternal and Child Health Certificate Registry of Liaoning Province. Controls were a random sample of healthy livebirths without any birth defects delivered in the selected five cities during 2010–2015. Ambient air monitoring data for PM_10_ were obtained from 75 monitoring stations in Liaoning Province. The multivariable logistic regression models were established to calculate adjusted odds ratios (OR) and 95% confidence intervals (CI). We further performed sensitivity analyses by using three propensity score methods. A total of 749 spina bifida cases and 7,950 controls were included. After adjusting for potential confounders, spina bifida was associated with a 10 μg/m^3^ increment in PM_10_ during the first trimester of pregnancy (adjusted OR = 1.06, 95% CI: 1.00–1.12) and the 3 months before pregnancy (adjusted OR = 1.12, 95% CI: 1.06–1.19). The adjusted ORs in the final model for the highest vs. the lowest quartile were 1.51 (95% CI: 1.04–2.19) for PM_10_ during the first trimester of pregnancy and 2.01 (95% CI: 1.43–2.81) for PM_10_ during the 3 months before pregnancy. Positive associations were found between PM_10_ exposures during the single month exposure windows and spina bifida. Sensitivity analyses based on two propensity score methods largely reported similar positive associations. Our findings support the evidence that maternal PM_10_ exposure increases the risk of spina bifida in offspring. Further, validation with a prospective design and a more accurate exposure assessment is warranted.

## Introduction

Spina bifida is a birth defect characterized by failure of the embryonic neural tube to close, which leads to deformities of the spinal cord and vertebral column (1). Spina bifida tends to be more common in girls (2), and prevalence rates vary greatly depending on geographical location (1). The summary prevalence of spina bifida was highest in Asia (243.14 per 100,000) and lowest in North America (38.70 per 100,000) in the meta-analysis reporting on live births, stillbirths, and terminations of pregnancy (3). This phenomenon may originate from discrepancies in race/ethnicity as well as preventive policies, and environmental factors might play a part in progression of this malformation (4). The etiology of spina bifida, including chromosome abnormalities, single gene disorders, and teratogenic exposures, is heterogeneous (2). Several risk factors associated with spina bifida have been identified, including inadequate maternal intake of folic acid (5) and pregestational maternal diabetes (6). Given that embryonic maldevelopment resulting in birth defects is a multifactorial process (7), it is important to identify modifiable environmental factors.

Air pollution is the biggest environmental risk factor of human health, resulting in more than 4 million deaths annually due to respiratory diseases in the world (8). Particulate matter (PM) is one of the most prevalent air pollutants, and many studies have reported a direct association between exposure to PM and negative health impacts (8). A number of epidemiological studies have also demonstrated positive associations between maternal PM exposure during pregnancy and adverse birth outcomes, such as preterm birth (9), low birth weight (10), and birth defects (11). A recent meta-analysis (12) on ambient air pollution and cardiac anomalies reported that each 10 μg/m^3^ increment in PM_10_ is associated with increased risk of atrial septal defects. However, there has been conflicting evidence of the effect of maternal PM_10_ exposure during pregnancy on certain types of birth defects because of great variability in the study populations, sample sizes, exposure assessments, ascertainment methods, and statistical adjustments. The association of ambient air pollution with spina bifida has not been well-established because of lack of sufficient evidence. To date, we have found only two studies (13, 14) with small sample sizes reporting the association of PM_10_ exposure during pregnancy and spina bifida risk, and the results were non-significant. Uncertainties remain regarding the aforementioned association.

Air pollution in China has received increasing attention in recent years due to its high levels and long duration (15). Specifically, air pollution in northern China is generally considered to be worse than that in southern China, which may be related to unique topographic features, climatic characteristics, and emissions sources (16). Industry plays an extremely important role in the economic development of Liaoning Province, accompanied by serious air pollution. A previous national study reported that the annual population-weighted-average values of PM_10_ in Liaoning Province from 2014 to 2016 were 101.3, 92.7, and 79.9 μg/m3, respectively, which exceeded the recommended annual PM_10_ concentration limit of 70 μg/m3 (17). Given the high prevalence of spina bifida and the high level of PM_10_ exposure in Liaoning Province, a further investigation is warranted. Therefore, we established a population-based case-control study to determine the association between maternal PM_10_ exposure and the risk of spina bifida using a 6-year accumulated data.

## Materials and Methods

### Study Population

Liaoning Province, located in the northeast of China, is our study area with an area of 148,000 km^2^ and a population of nearly 43 million. The study population included all livebirths, stillbirths, and induced abortions enrolled within the Maternal and Child Health Certificate Registry of Liaoning Province between 1 January 2010 and 31 December 2015. A detailed description of the registry is available in our previous studies (18–20). In short, this birth registry throughout the whole province was set up in 1988 and monitored nearly 6,000 cases of birth defects per year during the study period. Liaoning Province is one of the 31 provinces in China that establish a population-based active surveillance system and is required to submit surveillance data to the Chinese Birth Defects Surveillance Network (21, 22).

We identified all spina bifida cases (livebirths, stillbirths, and terminations of pregnancy following prenatal diagnosis) from the registry between 2010 and 2015. Spina bifida (International Classification of Diseases, 10th, Clinical Modification code Q05) was diagnosed by clinical and imaging examinations until the end of infancy. The selection of unaffected controls has been reported in full (19, 23). Briefly, we divided Liaoning Province into three geographical regions and selected healthy livebirths without any birth defects born in five cities (Shenyang, Dalian, Fuxin, Chaoyang, and Huludao) in three regions as the source of controls based on the birth population proportion, which can well-cover the province's different degrees of air pollution and economic development. In this study, controls were a random sample, representing 1.5% of livebirths born in the above five cities between 2010 and 2015.

### Data Collection

The data collection process of the registry has been described in detail (19, 21, 24). In brief, a three-level (county, province, and central) surveillance network as well as corresponding expert groups were set up to deal with daily data collection. At participating hospitals, relevant information was collected by interview with the mothers of newborns (or aborted fetuses) with spina bifida using a birth defects registration form. We screened the maternal information during the data collection process to ensure that there was no duplication of enrollment. When the mother gave birth again during the study period, we only included the information from her first enrollment interview. Based on the Chinese Maternal and Child Health Surveillance Workbook, the determination of birth defects and the quality of data on birth defects were reviewed by experts at all levels from surveillance networks. All data were finally reported to the provincial maternal and child health institution through a step-by-step submission process. Furthermore, an independent retrospective validation was conducted by a panel of national-level clinical experts (25).

### Exposure Assessment

**Figure 1:**
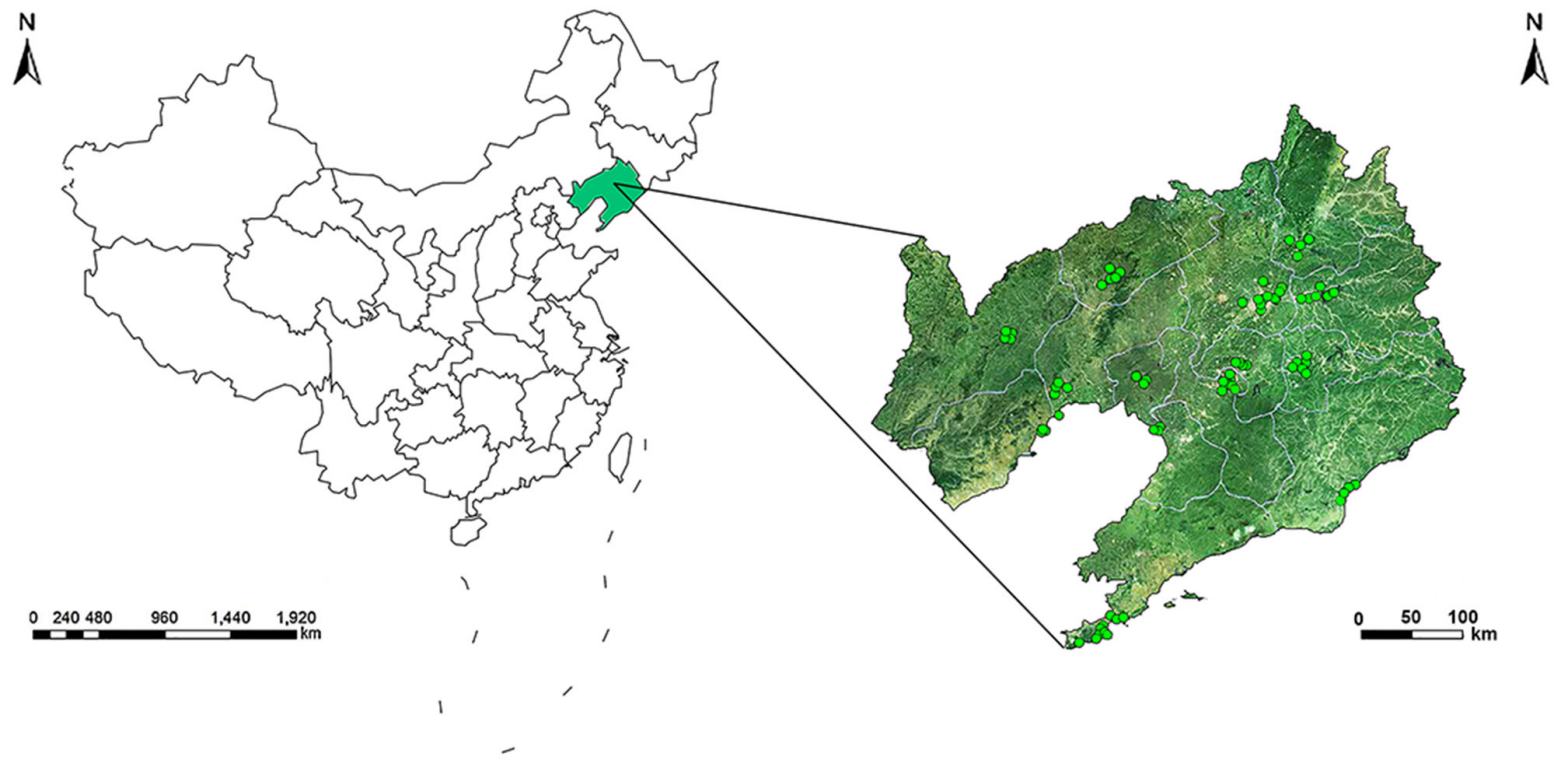
Geographical location of 75 air monitoring stations in 14 cities of Liaoning Province.

The monthly average values of air pollutants of 14 cities in Liaoning Province during 2010–2015 were measured using the daily ambient air pollution monitoring data from 75 monitoring stations (Figure 1) in Liaoning Province. The monthly mean air pollutant concentrations from all monitoring stations of each city were integrated for an average for each mother in corresponding city. In this study, we treated the 1st trimester, the 1st, 2nd, and 3rd month after conception, the 3 months before conception, and the 1st, 2nd, and 3rd month before conception as the exposure windows of interest. The conception date was defined as the first day of last menstrual period according to the previous study (26). If the date of conception falls in the first half of a month, the month is defined as the first month after conception. If the date of conception falls in the second half of a month, the month is defined as the first month before conception.

### Statistical Analyses

Categorical (continuous) variables were expressed as counts and corresponding percentages (median and interquartile range [IQR]), and intergroup comparisons were analyzed using the chi-square test (Mann-Whitney *U*-test). The monthly and seasonal average PM_10_ concentrations during 2010–2015 were presented aiming to provide a set of multiperspective panoramas of ambient air pollution of Liaoning Province.

We used adjusted odds ratios (OR) and 95% confidence intervals (CI) as measures of associations between developmental period-specific PM_10_ exposures and spina bifida. We selected covariates (maternal age [<20, 20–24, 25–29, 30–34, ≥35], sex [female/male], season of conception [spring, summer, autumn, winter], gravidity [<2/≥2], parity [0, 1, ≥2], maternal education [elementary school or less, middle school, high school, college, or above], and maternal SO_2_ and NO_2_ exposures [continuous] in the same exposure window) a priori based on previous literature (27–30) and data availability. Gravidity is defined as the total number of pregnancies and parity is defined as the total number of live births. For model 1, maternal SO_2_ and NO_2_ exposures, and PM_10_ exposure were added to the multivariable model. Then, selected covariates, including maternal age, sex, season of conception, gravidity, parity, and maternal education, were further added to the multivariable model (model 2). PM_10_ exposures were evaluated both as a continuous variable (per 10 μg/m^3^ increment) and quartiles using the distribution among the entire study population. We assessed the statistical significance for a linear trend through fitting a continuous variable (P_12.5_, P_37.5_, P_67.5_, P_87.5_ on the basis of the distribution among the entire study population) in the model (31).

We estimated propensity score by fitting a multivariable logistic regression model with all covariates included in the main analysis except for maternal SO_2_ and NO_2_ exposures and further performed sensitivity analyses using three propensity score methods. First, a 1:1 nearest-neighbor matching was conducted between cases and controls using a caliper width equal to 0.2 of the standard deviation of the logit of the propensity score (32). In the propensity score-matched subset, a multivariable logistic model adjusted for maternal SO_2_ and NO_2_ exposures was used to assess the association of maternal PM_10_ exposure with spina bifida risk. A second sensitivity analysis was conducted using an inverse probability weighted logistic regression model. Standardized mean differences were calculated to quantify the balance of covariates between cases and controls after matching and weighting, with a value <0.1 representing an adequate balance (33). Third, we included the propensity score as an additional covariate in the final multivariable logistic regression model (34).

The statistical analyses were done using SAS version 9.4 and R version 4.0.5. Statistical significance was set at *p* < 0.01 and based on the two-sided test.

## Results

**Figure 2:**
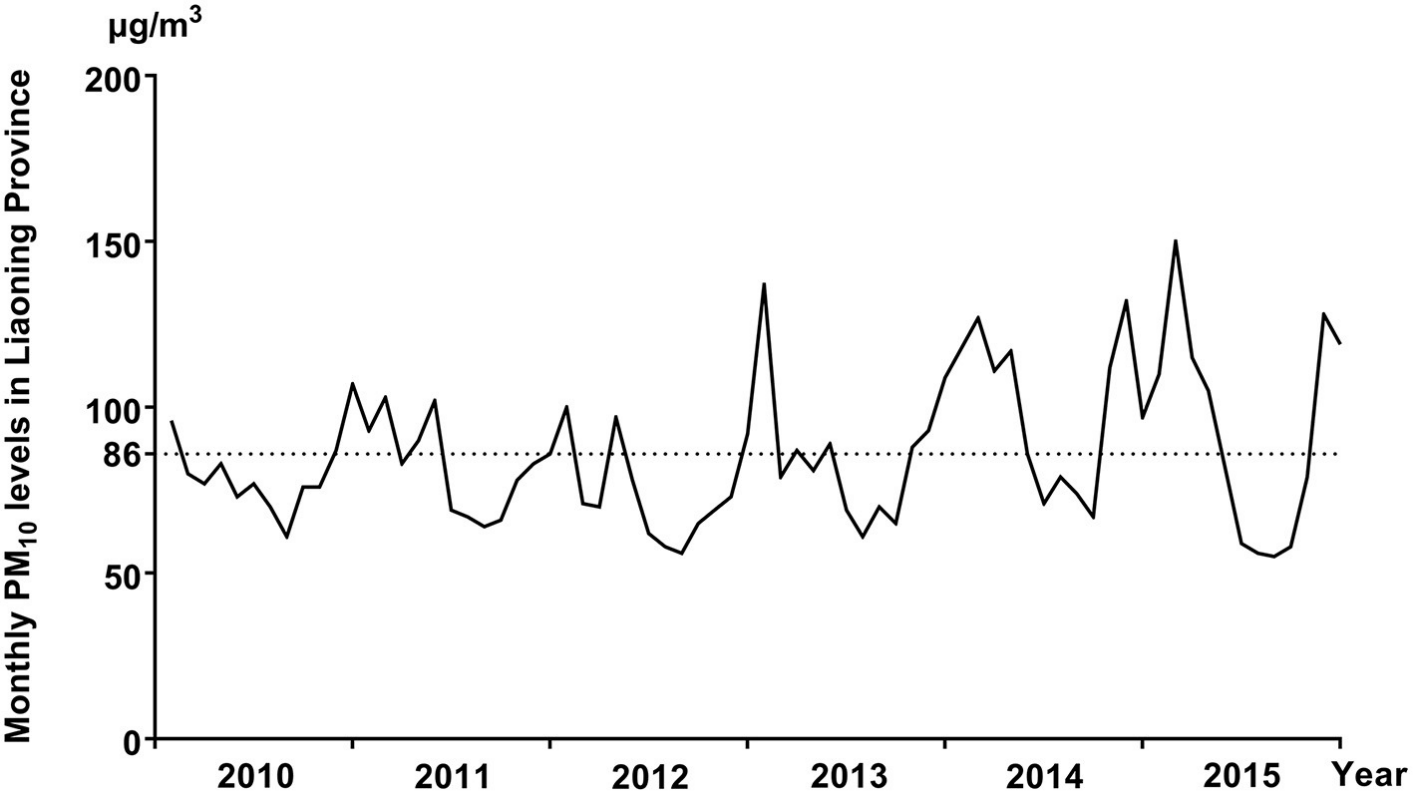
The monthly mean concentrations of PM_10_ in entire Liaoning Province, between 2010 and 2015.

**Figure 3:**
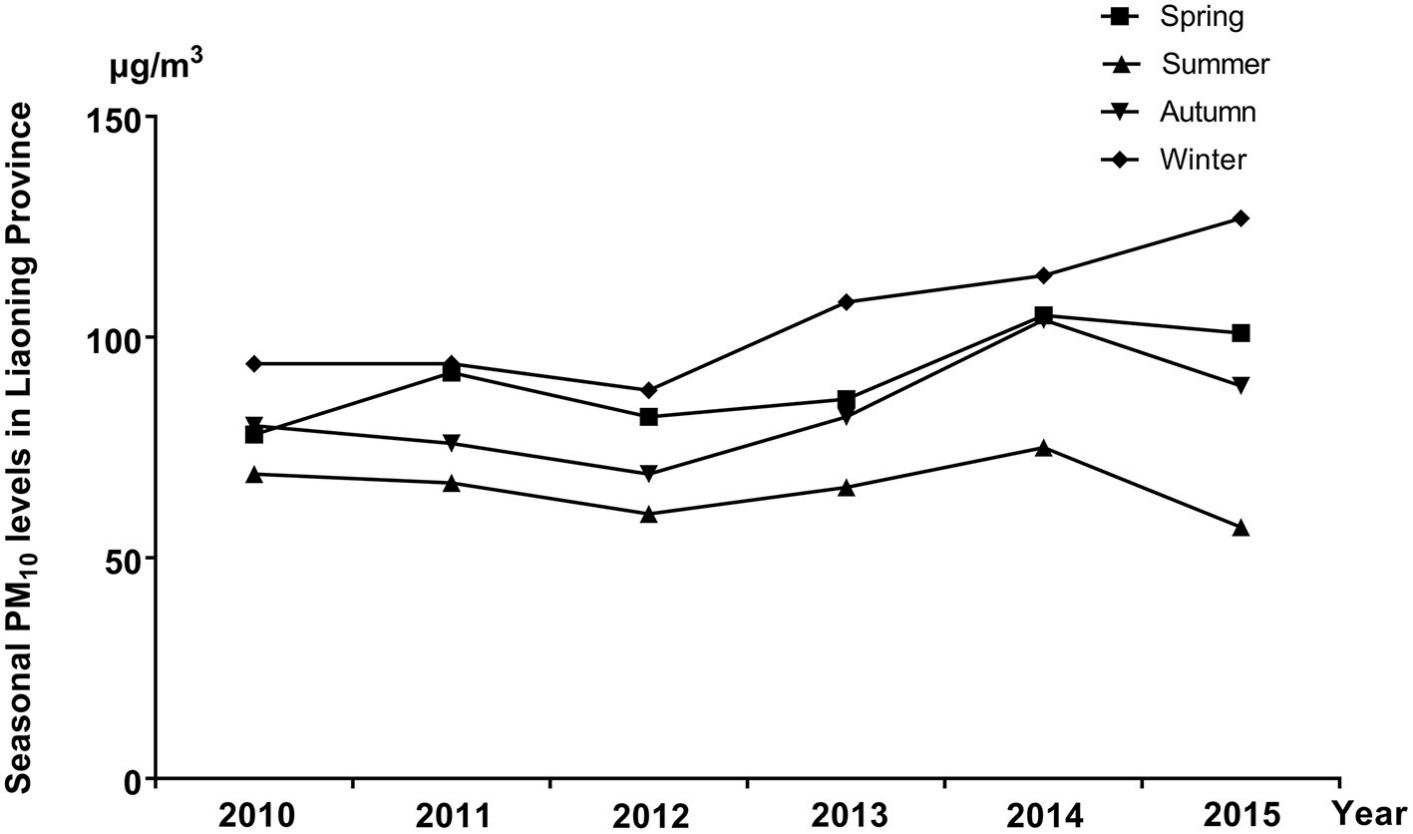
The seasonal mean concentrations of PM_10_ in entire Liaoning Province, between 2010 and 2015.

**Figure 4:**
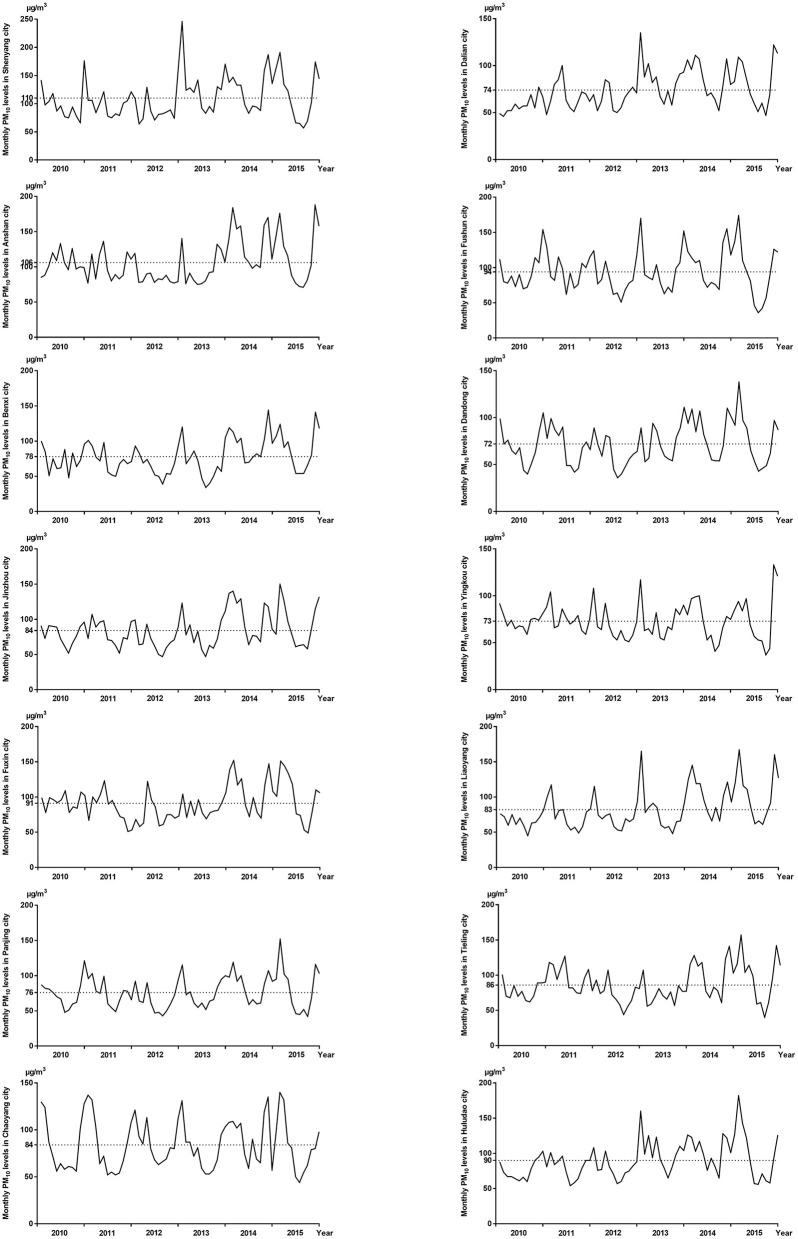
The monthly mean concentrations of PM_10_ in 14 cities of Liaoning Province, between 2010 and 2015.

The distribution of selected characteristics among spina bifida cases (*n* = 749) and healthy controls (7,950) without any birth defects is shown in Table 1. The median maternal age, gestational age, and birth weight of cases were significantly lower than controls. A larger proportion of spina bifida cases was female and had season of conception in autumn and winter than controls. Mothers of spina bifida cases were more likely to be less educated, and to have higher gravidity and parity compared with counterparts. The monthly mean concentrations of PM_10_ in entire Liaoning Province continued to fluctuate during 2010–2015, with a 6-year average level of 86 μg/m3 (Figure 2). During the study period, the most serious ambient PM air pollution (PM_10_) in Liaoning Province occurred in winter, while the average concentration of PM_10_ was lowest in summer (Figure 3). In addition, Shenyang's ambient PM air pollution was worse than 13 other cities in Liaoning Province (Figure 4). Table 2 presents the air pollution exposure estimates during different time periods for cases and controls. The spina bifida cases and healthy controls were exposed to different concentrations of PM_10_ within the same exposure window, though, there were small differences between the two groups.

**Table 1:** General characteristics of the study population.

**Characteristics**	**Cases**	**Controls**	***P*-value**
	**(*n* = 749)**	**(*n* = 7,950)**	
Maternal age, years	27 (23–31)	29 (26–32)	<0.0001
<20	24 (3%)	53 (1%)	<0.0001
20–24	227 (30%)	1,090 (14%)	
25–29	260 (35%)	3,561 (45%)	
30–34	147 (20%)	2,297 (29%)	
≥35	91 (12%)	949 (12%)	
Gender			0.036
Female	400 (53%)	3,927 (49%)	
Male	349 (47%)	4,023 (51%)	
Season of conception			<0.001
Spring	195 (26%)	2,106 (26%)	
Summer	191 (26%)	2,829 (36%)	
Autumn	177 (24%)	1,705 (21%)	
Winter	186 (25%)	1,310 (16%)	
Gestational age, weeks	25 (21–31)	39 (38–40)	<0.001
<37	614 (82%)	257 (3%)	
≥37	135 (18%)	7,693 (97%)	
Birth weight, grams	800 (500–2,000)	3,400 (3,130–3,700)	<0.001
<2,500	593 (79%)	174 (2%)	
2,500–<4,000	145 (19%)	6,840 (86%)	
≥4,000	11 (1%)	936 (12%)	
Gravidity			0.002
<2	431 (58%)	5,026 (63%)	
≥2	318 (42%)	2,924 (37%)	
Parity			<0.001
0	328 (44%)	5,931 (75%)	
1	339 (45%)	1,764 (22%)	
≥2	82 (11%)	255 (3%)	
Maternal education			<0.001
Elementary school or less	55 (7%)	265 (3%)	
Middle school	444 (59%)	2,912 (37%)	
High school	144 (19%)	1,723 (22%)	
College or above	106 (14%)	3,050 (38%)	

*Data are median (IQR) or n (%). p-values were calculated by Mann-Whitney U-test or χ^2^ test, as appropriate*.

**Table 2:** Summary statistics of participants' exposure to air pollutants (μg/m^3^) in different time periods.

**Air pollutants**	**Exposure windows**	**Cases (*****n*** **=** **749)**		**Controls (*****n*** **=** **7,950)**	
		**Median (IQR)**	**Range**	**Median (IQR)**	**Range**
PM_10_	After conception				
	0–1 month	82 (69–102)	36–246	82 (67–105)	48–246
	1–2 month	84 (69–103)	34–246	82 (67–103)	48–246
	2–3 month	83 (69–104)	34–246	83 (69–107)	48–246
	0–3 month	86 (72–102)	40–177	87 (68–106)	52–177
	Before conception				
	0–1 month	85 (68–102)	44–187	85 (71–106)	48–246
	1–2 month	81 (67–101)	40–246	85 (71–107)	46–246
	2–3 month	81 (67–99)	34–246	86 (71–108)	46–246
	0–3 month	85 (70–101)	41–177	89 (75–105)	49–177
SO_2_	After conception, 0–3 month	37 23–62	6–201	30 (21-65)	7–201
	Before conception, 0–3 month	38 (23–60)	6–201	34 (23–59)	7–201
NO_2_	After conception, 0–3 month	34 (27–39)	9–64	35 (31–42)	16–64
	Before conception, 0–3 month	34 (26–40)	10–61	36 (31–42)	17–64

Table 3 shows the associations between maternal PM_10_ exposures during various exposure windows and the risk of spina bifida from the three-pollutant and fully adjusted models. Overall, in the three-pollutant model, there were no significant associations of developmental period-specific PM_10_ exposures with spina bifida using PM_10_ as both a categorical and continuous variable. After multivariable adjustment, we found a 6–12% increase in the odds of spina bifida per 10 μg/m3 increment in PM_10_ exposures during different time periods except for the 3rd month before conception. In addition, effect estimates for the highest vs. the lowest quartile ranged from 1.51 (1.04–2.19) to 2.23 (1.60–3.09) for maternal PM_10_ exposure in different exposure windows. Notably, the strongest associations of maternal PM_10_ exposures with spina bifida tended to be found in the third quartile, between 82 and 107 μg/m3.

**Table 3:** Odds ratios and 95% confidence intervals for spina bifida by maternal exposure quartiles of PM_10_ of different exposure windows.

**Exposure windows**	**Quartiles of PM**_**10**_ **exposure (μg/m**^**3**^**)**				***P*-value for trend**	**Continuous (per 10 μg/m^**3**^ increment); *P*-value**
	**1**	**2**	**3**	**4**		
After conception, 0–1 month	<67	≥67–<82	≥82–<104	≥104		
Cases/controls	162/1,945	204/1,754	204/2,245	179/2,006		
Model 1^*^	1.00	1.49 (1.20–1.86)	1.52 (1.21–1.91)	1.29 (0.96–1.74)	0.06	1.00 (0.96–1.04); 0.93
Model 2^*^	1.00	1.86 (1.45–2.38)	2.51 (1.93–3.29)	2.02 (1.44–2.84)	<0.01	1.07 (1.02–1.11); <0.01
After conception, 1–2 month	<68	≥68–<82	≥82–<103	≥103		
Cases/controls	166/1,994	182/1,910	211/2,030	190/2,016		
Model 1^*^	1.00	1.23 (0.98–1.53)	1.69 (1.36–2.12)	1.59 (1.19–2.12)	<0.01	1.01 (0.97–1.05); 0.61
Model 2^*^	1.00	1.71 (1.34–2.20)	2.92 (2.25–3.81)	2.23 (1.60–3.09)	<0.01	1.06 (1.01–1.10); <0.01
After conception, 2–3 month	<69	≥69–<83	≥83–<107	≥107		
Cases/controls	184/1,878	179/2,009	213/1,994	173/2,069		
Model 1^*^	1.00	1.01 (0.81–1.26)	1.53 (1.22–1.91)	1.31 (0.99–1.74)	<0.01	1.00 (0.97–1.04); 0.98
Model 2^*^	1.00	1.56 (1.23–1.99)	2.06 (1.59–2.68)	1.65 (1.19–2.28)	<0.01	1.03 (0.99–1.07); 0.20
After conception, 0–3 month	<68	≥68–<87	≥87–<106	≥106		
Cases/controls	145/1,852	230/2,055	223/1,941	151/2,102		
Model 1^*^	1.00	1.34 (1.08–1.67)	1.58 (1.25–2.00)	1.01 (0.72–1.41)	0.26	0.97 (0.92–1.03); 0.34
Model 2^*^	1.00	2.00 (1.56–2.56)	2.32 (1.75–3.08)	1.51 (1.04–2.19)	<0.01	1.06 (1.00–1.12); 0.06
Before conception, 0–1 month	<71	≥71–<85	≥85–<106	≥106		
Cases/controls	214/1,953	160/1,905	206/2,039	169/2,053		
Model 1^*^	1.00	0.83 (0.66–1.03)	1.46 (1.17–1.82)	0.98 (0.74–1.29)	0.23	0.98 (0.94–1.01); 0.21
Model 2^*^	1.00	1.28 (1.01–1.64)	2.00 (1.55–2.60)	1.70 (1.23–2.36)	<0.01	1.06 (1.01–1.10); 0.012
Before conception, 1–2 month	<69	≥69–<85	≥85–<107	≥107		
Cases/controls	207/1,870	203/1,719	186/2,264	153/2,097		
Model 1^*^	1.00	1.13 (0.92–1.39)	1.08 (0.87–1.36)	0.89 (0.67–1.16)	0.51	0.96 (0.93–1.00); 0.05
Model 2^*^	1.00	1.98 (1.57–2.51)	1.63 (1.27–2.10)	1.80 (1.32–2.45)	<0.01	1.07 (1.03–1.12); <0.01
Before conception, 2–3 month	<71	≥71–<85	≥85–<107	≥107		
Cases/controls	230/1,937	177/1,563	211/2,334	131/2,116		
Model 1^*^	1.00	1.06 (0.86–1.31)	1.20 (0.97–1.49)	0.82 (0.62–1.09)	0.56	0.98 (0.94–1.02); 0.25
Model 2^*^	1.00	1.83 (1.43–2.32)	2.07 (1.62–2.64)	1.90 (1.38–2.62)	<0.01	1.11 (1.06–1.15); <0.01
Before conception, 0–3 month	<73	≥73–<88	≥88–<105	≥105		
Cases/controls	232/1,770	171/1,969	188/2,055	158/2,156		
Model 1^*^	1.00	0.71 (0.57–0.87)	0.97 (0.78–1.20)	0.87 (0.64–1.17)	0.48	0.92 (0.87–0.97); <0.01
Model 2^*^	1.00	1.24 (0.97–1.58)	1.74 (1.34–2.26)	2.01 (1.43–2.81)	<0.01	1.12 (1.06–1.19); <0.01

* *Model 1: adjusted for maternal SO_2_ and NO_2_ exposures (continuous) in the same exposure window. Model 2: as for model 1 and additionally adjusted for maternal age (<20, 20–24, 25–29, 30–34, ≥35), sex (female/male), season of conception (spring, summer, autumn, winter), gravidity (<2/≥2), parity (0, 1, ≥2), and maternal education (elementary school or less, middle school, high school, college or above)*.

**Figure 5:**
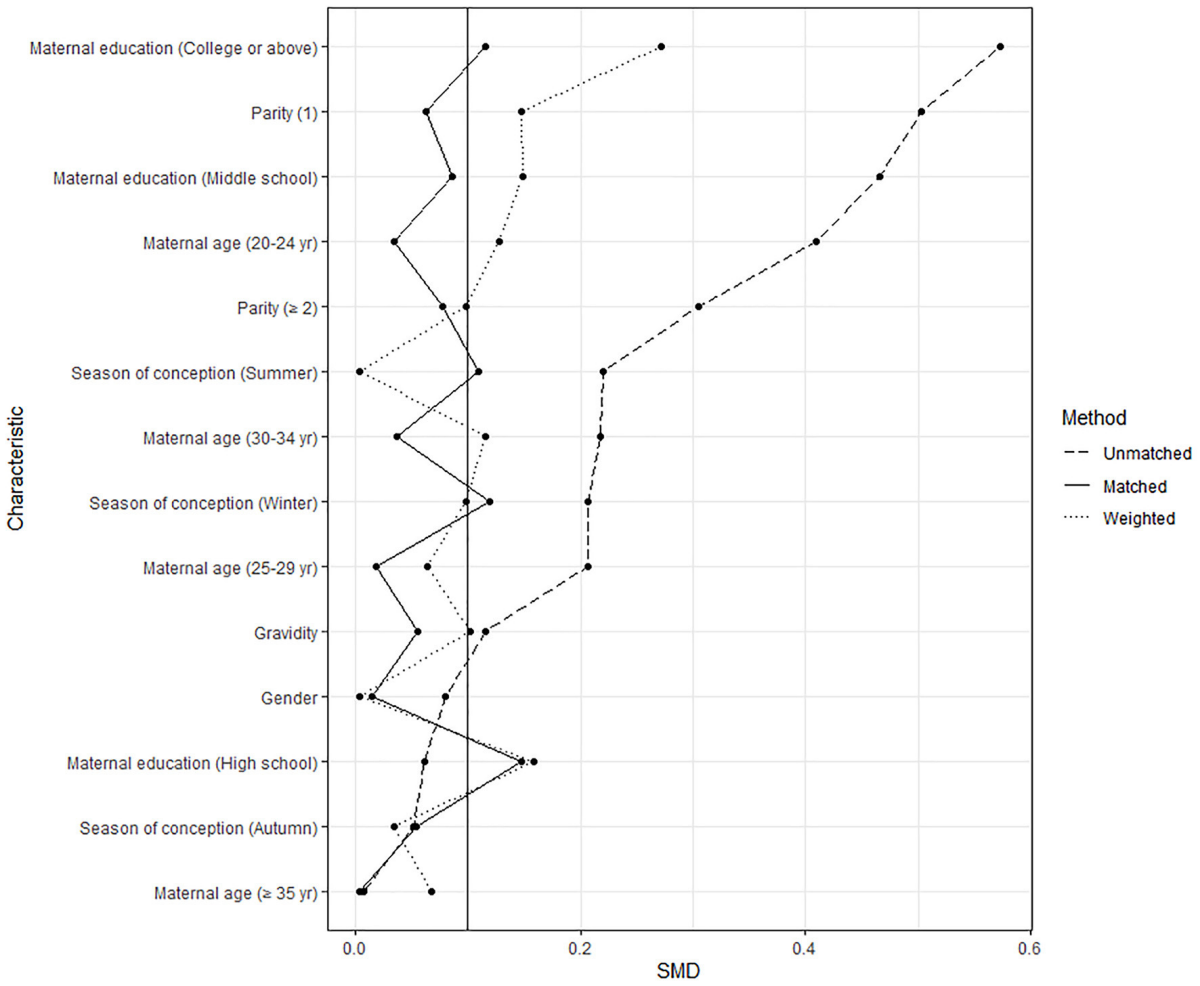
The values for standardized mean differences in the initial, matched, and weighted data.

The values for standardized mean differences in the initial, matched, and weighted data are presented in Figure 5. Most of characteristics had standardized mean difference values of more than 0.1 before matching, which represents a between-group imbalance. Matching and weighting resulted in a relative balance between spina bifida cases and controls on selected characteristics. Table 4 shows the associations between maternal PM_10_ exposures during different exposure windows and spina bifida risk in the propensity-score analyses. We generated a subset of 677 spina bifida cases and 677 matched controls using 1:1 propensity score matching. Propensity score-matched analysis based on continuous exposure variables presented positive associations of maternal PM_10_ exposures during all examined exposure windows with spina bifida risk, with point estimates ranging from 1.17 to 1.35. The results from multivariable propensity-score analyses were consistent with the primary findings. However, in the logistic regression with inverse probability weighting, no significant associations were observed between spina bifida risk and maternal PM_10_ exposures, except for PM_10_ during the second month after conception (OR = 1.05, 95% CI 1.01–1.08).

**Table 4:** Association of maternal PM_10_ exposure with spina bifida risk in the propensity-score analyses.

	Propensity-score analyses—odds ratio and 95% CI^*^		
	**With matching**^**†**^	**With inverse probability weighting**^**†**^	**Adjusted for propensity score**^**‡**^
**Cases/controls**	677/677	946/7,923	749/7,950
**After conception**			
0–1 month	1.17 (1.10–1.24)	1.01 (0.98–1.05)	1.06 (1.02–1.11)
1–2 month	1.19 (1.13–1.26)	1.05 (1.01–1.08)	1.05 (1.01–1.10)
2–3 month	1.18 (1.11–1.25)	0.99 (0.95–1.02)	1.03 (0.99–1.07)
0–3 month	1.24 (1.15–1.33)	1.01 (0.97–1.06)	1.06 (1.00–1.12)
**Before conception**			
0–1 month	1.13 (1.07–1.20)	1.02 (0.99–1.06)	1.05 (1.00–1.09)
1–2 month	1.24 (1.17–1.31)	0.99 (0.96–1.02)	1.07 (1.02–1.11)
2–3 month	1.33 (1.26–1.41)	0.97 (0.94–1.00)	1.10 (1.06–1.15)
0–3 month	1.35 (1.26–1.45)	0.97 (0.93–1.02)	1.11 (1.05–1.18)

* *Shown is the odds ratio for per 10 μg/m^3^ increment of maternal PM_10_ exposure*.

† *Adjusted for maternal SO_2_ and NO_2_ exposures (continuous) in the same exposure window*.

‡ *Adjusted for maternal SO_2_ and NO_2_ exposures (continuous) in the same exposure window, maternal age (<20, 20–24, 25–29, 30–34, ≥35), sex (female/male), season of conception (spring, summer, autumn, winter), gravidity (<2/≥2), parity (0, 1, ≥2), maternal education (elementary school or less, middle school, high school, college or above), and propensity score*.

## Discussion

This population-based case-control study examined the associations of maternal PM_10_ exposures during eight different exposure windows with the risk of spina bifida among offspring in Liaoning Province, China over a 6-year period. We found that developmental period-specific PM_10_ exposures were associated with an increased risk of spina bifida in this area. This study was currently the largest sample size study on the association between maternal PM_10_ exposure and spina bifida. The exact mechanism by which PM_10_ causes birth defects remains elusive, but several possible mechanisms have been postulated, such as placental inflammation (35), oxidative stress (36, 37), and alteration of molecular signaling (11).

To our knowledge, only two studies, conducted in Italy (13) and the United States (14), have described the association of maternal PM_10_ exposure with spina bifida risk. Maternal PM_10_ exposure varies greatly depending on geographical location, and the results of studies conducted in developed countries with relatively low levels of PM_10_ exposure may not be applicable to some heavily polluted areas. An Italian case-control study (13) recruited 228 cases of birth defects and 228 matched healthy newborns, and used a dispersion model to evaluate maternal PM_10_ exposure during the first trimester of pregnancy. The Italian study reported a non-significant association between a 1 μg/m^3^ increment in PM_10_ during early pregnancy and spina bifida risk. Compared with our study, its main limitation is the small sample size, which may increase the statistical inaccuracy. In a case-control study (14) of 8 counties in the United States, the adjusted OR for the highest quartile vs. the lowest quartile was increased in relation to maternal PM_10_ exposure during the first 2 months after conception, although, not statistically significantly. In case-control studies, covariate information obtained from interviews may be subject to recall bias. In addition, compared to cohort studies, our study was unable to draw a causal relationship.

A previous review of ambient PM air pollution and birth defects emphasized that the toxicity of PM is the result of the combined effect of PM and other toxic substances because of the strong adsorption of PM (11). Adsorbed toxic substances, such as persistent organic pollutants and heavy metals, may be responsible for the associations observed in the air pollution studies. A case-control study (38) in Texas showed that exposure to benzene was positively associated with the risk of spina bifida. Texas's ambient levels of benzene rank first in the United States (39), therefore, this positive association may not be replicated in our study area. However, this is an inevitable question in studies that assessed the impacts of air pollutants on birth defects, and further, efforts are needed to explore the independent effects. In addition, regional differences in disease diagnosis may exist in multicenter studies. In our study, we included cases of spina bifida diagnosed from different participating hospitals in 14 cities in Liaoning Province during the study period, so variations in ascertainment methods were difficult to avoid. Unlike easily detectable birth defects, such as limb defects, the diagnosis of spina bifida may be more complicated. However, several quality control measures taken during the case collection process can correct diagnostic errors to some extent. The association between PM_10_ estimates and spina bifida appears to be non-linear. For some exposure windows, the highest effect estimates were observed for PM_10_ exposure in the 3rd quartile, whereas, the effect estimates were reduced for exposure to PM_10_ in the fourth quartile. A possible explanation is that women in highly polluted areas spend less time outdoors during pregnancy, which leads to overestimation of PM_10_ exposure levels of mothers in the fourth quartile.

A major advantage of our study is the large sample size, which allows us to explore the associations of interest in a more statistically precise manner. Another advantage is that the exposure windows are comprehensive, from the third month before conception to the third month after conception. It is worth noting that exposure to air pollutants before pregnancy has rarely been studied. In line with our findings, two previous studies (7, 40) in the United States have shown that exposure to higher levels of ambient PM before pregnancy increases the risk of birth defects. Women may need to take precautions against air pollution before they become pregnant.

Due to some limitations, our results need to be interpreted with caution. A main limitation was the imprecision of exposure assessment. In this study, we assigned the average PM_10_ concentration of all air monitoring stations in the city where the mother lived during pregnancy to each birth. This approach reduced the accuracy of exposure assessment, leading to exposure misclassification. Further, studies with a more accurate exposure assessment, such as dispersion or land-use regression models, are warranted (41). In addition, due to lack of data, we failed to take into account the exposures of gravidae in the microenvironments, such as indoor air pollution sources, workplace, and commuting, which may also lead to exposure misclassification. Differences in the exposures in the microenvironments may influence the association between ambient air pollution exposure and birth defects. A study (42) on exposure to indoor air pollution indicated that different cooking fuels and cooking times can cause different personal PM exposure. In the future, precise information on the exposures of gravidae in the microenvironments is worth collecting and adjusting in the statistical model. Second, we did not consider migration/mobility during pregnancy when assessing maternal PM_10_ exposures. Two previous large-scale studies (28, 43) in China reported that only 3% of mothers moved during pregnancy. A review of 14 studies also reported that overall mobility rates were 9–32% and highest in the second trimester (44). Therefore, measurement errors due to migration/mobility were unlikely to affect the evaluations of associations in our study. Another limitation of our study was lack of information on maternal diseases as well as nutritional status during pregnancy. Inadequate maternal folate intake and maternal diabetes may also increase the risk of spina bifida in offspring. However, these factors are unlikely to be related to ambient air pollution and may be partially compensated by adjusting maternal education level. Fourth, due to the unbalanced city selection between the control and case groups (5 vs. 14 cities), we failed to take into account the regional influence, which may affect the interpretation of study results. Finally, the inconsistent association between maternal PM_10_ exposure and spina bifida was observed in the propensity score-weighted sensitivity analysis. However, for our study, the propensity scores of most subjects were close to 0. Therefore, the results from the inverse probability weighting should be interpreted with caution.

## Conclusions

In conclusion, maternal PM_10_ exposures during the first trimester of pregnancy and the 3 months before conception may elevate the risk of spina bifida in offspring.

## Data Availability Statement

The raw data supporting the conclusions of this article will be made available by the authors, without undue reservation.

## Ethics Statement

The studies involving human participants were reviewed and approved by Liaoning Women and Childrenan Health Hospital. Written informed consent to participate in this study was provided by the participants' legal guardian/next of kin.

## Author Contributions

Y-HH and NL: study conceptualization, analytic strategy, and design. JL, SL, Y-LC, L-LL, and Z-JC: data collection. HL and C-ZJ: data cleaning and discrepancy checks. HL: analysis and interpretation of data. HL and Y-HH: manuscript preparation. All authors have read and approved the final manuscript.

## Conflict of Interest

The authors declare that the research was conducted in the absence of any commercial or financial relationships that could be construed as a potential conflict of interest.

## References

[1] Copp AJ Adzick NS Chitty LS Fletcher JM Holmbeck GN Shaw GM. Spina bifida. Nat Rev Dis Primers. (2015) 1:15007. 10.1038/nrdp.2015.7 27189655 PMC4898641

[2] Mitchell LE Adzick NS Melchionne J Pasquariello PS Sutton LN Whitehead AS. Spina bifida. Lancet. (2004) 364:1885–95. 10.1016/S0140-6736(04)17445-X 15555669

[3] Atta CA Fiest KM Frolkis AD Jette N Pringsheim T St GC. Global birth prevalence of spina bifida by folic acid fortification status: a systematic review and meta-analysis. Am J Public Health. (2016) 106:e24–34. 10.2105/AJPH.2015.302902 26562127 PMC4695937

[4] Copp AJ Adzick NS Chitty LS Fletcher JM Holmbeck GN Shaw GM. Spina bifida. Nat Rev Dis Primers. (2015) 1:15051. 10.1038/nrdp.2015.51 27189655 PMC4898641

[5] Martinez H Pachon H Kancherla V Oakley GP. Food fortification with folic acid prevents spina bifida and anencephaly: a need for paradigm shift in evidence evaluation for policy-Making. Am J Epidemiol. (2021) kwab061. 10.1093/aje/kwab061 33728445 PMC8485149

[6] Mcleod L Ray JG. Prevention and detection of diabetic embryopathy. Community Genet. (2002) 5:33–9. 10.1159/000064629 14960898

[7] Ren S Haynes E Hall E Hossain M Chen A Muglia L. Periconception exposure to air pollution and risk of congenital malformations. J Pediatr. (2018) 193:76–84. 10.1016/j.jpeds.2017.09.076 29237538 PMC5794608

[8] World Health Organization. Air Pollution. Available online at: https://www.who.int/health-topics/air-pollution#tab=tab_2 (accessed April 14, 2021)

[9] Zhao N Qiu J Zhang Y He X Zhou M Li M. Ambient air pollutant PM_10_ and risk of preterm birth in Lanzhou, China. Environ Int. (2015) 76:71–7. 10.1016/j.envint.2014.12.009 25553395 PMC4526148

[10] Arroyo V Diaz J Salvador P Linares C. Impact of air pollution on low birth weight in Spain: an approach to a National level study. Environ Res. (2019) 171:69–79. 10.1016/j.envres.2019.01.030 30660920

[11] Teng C Wang Z Yan B. Fine particle-induced birth defects: impacts of size, payload, and beyond. Birth Defects Res C Embryo Today. (2016) 108:196–206. 10.1002/bdrc.21136 27581067

[12] Hu CY Huang K Fang Y Yang XJ Ding K Jiang W. Maternal air pollution exposure and congenital heart defects in offspring: a systematic review and meta-analysis. Chemosphere. (2020) 253:126668. 10.1016/j.chemosphere.2020.126668 32278917

[13] Vinceti M Malagoli C Malavolti M Cherubini A Maffeis G Rodolfi R. Does maternal exposure to benzene and PM_10_ during pregnancy increase the risk of congenital anomalies? A population-based case-control study. Sci Total Environ. (2016) 541:444–50. 10.1016/j.scitotenv.2015.09.051 26410719 PMC4656073

[14] Padula AM Tager IB Carmichael SL Hammond SK Lurmann F Shaw GM. The association of ambient air pollution and traffic exposures with selected congenital anomalies in the San Joaquin Valley of California. Am J Epidemiol. (2013) 177:1074–85. 10.1093/aje/kws367 23538941 PMC3697063

[15] Ren Z Zhu J Gao Y Yin Q Hu M Dai L. Maternal exposure to ambient PM_10_ during pregnancy increases the risk of congenital heart defects: evidence from machine learning models. Sci Total Environ. (2018) 630:1–10. 10.1016/j.scitotenv.2018.02.181 29471186

[16] He J Gong S Yu Y Yu L Wu L Mao H. Air pollution characteristics and their relation to meteorological conditions during 2014-2015 in major Chinese cities. Environ Pollut. (2017) 223:484–96. 10.1016/j.envpol.2017.01.050 28122671

[17] Song C Wu L Xie Y He J Chen X Wang T. Air pollution in China: status and spatiotemporal variations. Environ Pollut. (2017) 227:334–47. 10.1016/j.envpol.2017.04.075 28482313

[18] Jiang YT Gong TT Zhang JY Huang YH Li J Liu S. Maternal exposure to ambient SO2 and risk of polydactyly and syndactyly: a population-based case-control study in Liaoning Province, China. Environ Sci Pollut Res Int. (2021) 28:11289–301. 10.1007/s11356-020-11351-5 33118065

[19] Zhang JY Gong TT Huang YH Li J Liu S Chen YL. Association between maternal exposure to PM_10_ and polydactyly and syndactyly: a population-based case-control study in Liaoning province, China. Environ Res. (2020) 187:109643. 10.1016/j.envres.2020.109643 32416360

[20] Zhang JY Wu QJ Huang YH Li J Liu S Chen YL. Association between maternal exposure to ambient PM_10_ and neural tube defects: a case-control study in Liaoning Province, China. Int J Hyg Environ Health. (2020) 225:113453. 10.1016/j.ijheh.2020.113453 31986338

[21] Gong TT Wu QJ Chen YL Jiang CZ Li J Li LL. Evaluating the time trends in prevalence of exomphalos in 14 cities of Liaoning province, 2006 to 2015. Sci Rep. (2016) 6:32901. 10.1038/srep32901 27604427 PMC5015066

[22] Huang YH Wu QJ Chen YL Jiang CZ Gong TT Li J. Trends in the prevalence of congenital hydrocephalus in 14 cities in Liaoning province, China from 2006 to 2015 in a population-based birth defect registry from the Liaoning Women and Children's Health Hospital. Oncotarget. (2018) 9:14472–80. 10.18632/oncotarget.24239 29581857 PMC5865683

[23] Liu FH Dai HX Gong TT Zhang JY Li J Chen ZJ. Maternal preconception and first trimester exposure to PM_10_ and the risk of oral clefts in offspring: a population-based, case-control study. Occup Environ Med. (2020) 77:721–7. 10.1136/oemed-2020-106434 32737151

[24] Xia J Huang YH Li J Liu S Chen YL Li LL. Maternal exposure to ambient particulate matter 10 mum or less in diameter before and after pregnancy, and anencephaly risk: a population-based case-control study in China. Environ Res. (2020) 188:109757. 10.1016/j.envres.2020.109757 32535358

[25] Xu L Li X Dai L Yuan X Liang J Zhou G. Assessing the trend of gastroschisis prevalence in China from 1996 to 2007 using two analytical methods. Birth Defects Res A Clin Mol Teratol. (2011) 91:177–84. 10.1002/bdra.20753 21308975

[26] Ji X Meng X Liu C Chen R Ge Y Kan L. Nitrogen dioxide air pollution and preterm birth in Shanghai, China. Environ Res. (2019) 169:79–85. 10.1016/j.envres.2018.11.007 30423521

[27] Huang CC Chen BY Pan SC Ho YL Guo YL. Prenatal exposure to PM_2.5_ and congenital heart diseases in Taiwan. Sci Total Environ. (2019) 655:880–6. 10.1016/j.scitotenv.2018.11.284 30481714

[28] Jin L Qiu J Zhang Y Qiu W He X Wang Y. Ambient air pollution and congenital heart defects in Lanzhou, China. Environ Res Lett. (2015) 10:074005. 10.1088/1748-9326/10/7/074005 31555342 PMC6760856

[29] Marshall EG Harris G Wartenberg D. Oral cleft defects and maternal exposure to ambient air pollutants in New Jersey. Birth Defects Res A Clin Mol Teratol. (2010) 88:205–15. 10.1002/bdra.20650 20146378 PMC2862481

[30] Ritz B Yu F Fruin S Chapa G Shaw GM Harris JA. Ambient air pollution and risk of birth defects in Southern California. Am J Epidemiol. (2002) 155:17–25. 10.1093/aje/155.1.17 11772780

[31] Liu R Young MT Chen JC Kaufman JD Chen H. Ambient air pollution exposures and risk of Parkinson disease. Environ Health Perspect. (2016) 124:1759–65. 10.1289/EHP135 27285422 PMC5089873

[32] Austin PC. Balance diagnostics for comparing the distribution of baseline covariates between treatment groups in propensity-score matched samples. Stat Med. (2009) 28:3083–107. 10.1002/sim.3697 19757444 PMC3472075

[33] Austin PC. An introduction to propensity score methods for reducing the effects of confounding in observational studies. Multivariate Behav Res. (2011) 46:399–424. 10.1080/00273171.2011.568786 21818162 PMC3144483

[34] Cheung KS Chan EW Chen L Seto WK Wong ICK Leung WK. Diabetes increases risk of gastric cancer after helicobacter pylori eradication: a territory-wide study with propensity score analysis. Diabetes Care. (2019) 42:1769–75. 10.2337/dc19-0437 31296646

[35] Kannan S Misra DP Dvonch JT Krishnakumar A. Exposures to airborne particulate matt er and adverse perinatal outcomes: a biologically plausible mechanistic framework for exploring potential effect modification by nutrition. Environ Health Perspect. (2006) 114:1636–42. 10.1289/ehp.9081 17107846 PMC1665414

[36] Kampa M Castanas E. Human health effects of air pollution. Environ Pollut. (2008) 151:362–7. 10.1016/j.envpol.2007.06.012 17646040

[37] Slama R Darrow L Parker J Woodruff TJ Strickland M Nieuwenhuijsen M. Meeting report: atmospheric pollution and human reproduction. Environ Health Perspect. (2008) 116:791–8. 10.1289/ehp.11074 18560536 PMC2430236

[38] Lupo PJ Symanski E Waller DK Chan W Langlois PH Canfield MA. Maternal exposure to ambient levels of benzene and neural tube defects among offspring: Texas, 1999-2004. Environ Health Perspect. (2011) 119:397–402. 10.1289/ehp.1002212 20923742 PMC3060005

[39] US. Environmental Protection Agency. Access the Air Quality System Data Mart. Available online at: http:// www.epa.gov/ttn/airs/aqsdatamart/access.htm (accessed April 14, 2021).

[40] Zhu Y Zhang C Liu D Grantz KL Wallace M Mendola P. Maternal ambient air pollution exposure preconception and during early gestation and offspring congenital orofacial defects. Environ Res. (2015) 140:714–20. 10.1016/j.envres.2015.06.002 26099933 PMC4498658

[41] Vrijheid M Martinez D Manzanares S Dadvand P Schembari A Rankin J. Ambient air pollution and risk of congenital anomalies: a systematic review and meta-analysis. Environ Health Perspect. (2011) 119:598–606. 10.1289/ehp.1002946 21131253 PMC3094408

[42] Jiang R Bell ML. A comparison of particulate matter from biomass-burning rural and non-biomass-burning urban households in northeastern China. Environ Health Perspect. (2008) 116:907–14. 10.1289/ehp.10622 18629313 PMC2453159

[43] Huang CC Wen HJ Chen PC Chiang TL Lin SJ Guo YL. Prenatal air pollutant exposure and occurrence of atopic dermatitis. Br J Dermatol. (2015) 173:981–8. 10.1111/bjd.14039 26202732

[44] Bell ML Belanger K. Review of research on residential mobility during pregnancy: consequences for assessment of prenatal environmental exposures. J Expo Sci Environ Epidemiol. (2012) 22:429–38. 10.1038/jes.2012.42 22617723 PMC3543155

